# Forest-EMCBE: an evolutionary ensemble learning algorithm for multiclass diagnosis of bacterial pneumonia using the CBC dataset

**DOI:** 10.3389/fbinf.2026.1792643

**Published:** 2026-03-18

**Authors:** Yimin Shen, Xiaotian Xu, Xiaoxi Hao, Cuimin Sun, Wei Lan

**Affiliations:** 1 School of Computer, Electronics and Information, Guangxi University, Nanning, China; 2 Qixia People’s Hospital of Shandong Province, Yantai, China; 3 Guangxi Colleges and Universities Key Laboratory of Multimedia Communications and Information Processing, Nanning, China

**Keywords:** bacterial pneumonia prediction, ECOC, ensemble learning, genetic algorithm, multiclass imbalance

## Abstract

**Introduction:**

Rapid diagnosis of bacterial pneumonia is crucial for clinical diagnosis and treatment, but traditional methods are time-consuming. The wide application of machine learning techniques in medical diagnosis provides an effective way to solve this problem. However, the complexity of medical datasets and the problem of class imbalance poses serious challenges to classical machine learning algorithms.

**Methods:**

Aiming at the multiclass imbalanced problem in complete blood count (CBC) datasets, this study proposes a novel ensemble learning algorithm, Forest of Evolutionary Multi-Classifiers Based on Bagging with Error-Correcting Output Coding (Forest-EMCBE). The algorithm integrates Multi-Objective Genetic Algorithm, Error-Correcting Output Codes (ECOC), and balanced sampling strategy, which enhances the generalization ability of the classifiers through a three-layer integrated structure.

**Results:**

To validate the effectiveness of the proposed method, we trained the diagnostic model on a CBC dataset, which contains 1,457 samples and 4 different classes of bacterial pneumonia results, and compared it with 11 state-of-the-art algorithms. The experimental results demonstrate the superior performance of the Forest-EMCBE algorithm on the CBC dataset, outperforming all other compared algorithms.

**Discussion:**

Based on the Shapley value-based feature importance analysis method, this study dissects the contributions of key features to the prediction outcomes and further elucidates the differential impacts of features such as age, gender, and neutrophil percentage on predicting infections by different bacterial species.

## 1 Introduction

Antimicrobial-resistant bacterial infections are a major threat to global public health, especially in low- and middle-income countries, where insufficient microbiological detection capacity leads to a paucity of relevant data. This makes the development of rapid and accurate pathogen detection technologies particularly urgent (Dunachie et al., 2020). In recent years, machine learning has been widely used in the biomedical field (Rajpurkar et al., 2022), which has opened up new avenues for the diagnosis of bacterial pneumonia by analyzing biomarkers such as leukocyte counts, neutrophil, and other biomarkers in the blood of post-infected patients, which can be helpful to the rapid diagnosis of the causative bacterial species.

However, constructing diagnostic models based on CBC datasets faces severe challenges due to the inherent class imbalanced property of healthcare data, which can severely constrain the performance of classical machine learning algorithms that optimize for overall accuracy and are prone to classification bias in highly imbalance data (Haixiang et al., 2017). In the real world, the problem of class imbalance in data exists not only in the field of medical diagnosis (Roy et al., 2023), but also widely in other fields, such as biology (Anand et al., 2010), error detection (Pan et al., 2023), and image recognition (Jin et al., 2022). To address this problems, a variety of approaches have been proposed by many researchers, which can be broadly categorized into data-level and algorithm-level approaches. The former includes resampling techniques including methods such as SMOTE (Chawla et al., 2002) and ADASYN (He et al., 2008); the latter can be categorized into cost-sensitive learning (Turney, 1994) and ensemble learning (Khan et al., 2024).

In recent years, ensemble learning has gained widespread application in addressing classification problems, demonstrating exceptional performance, particularly in tackling class imbalanced problems. GASIS was proposed for base classifier selection in ensemble learning, which effectively improves the performance of ensemble models by optimizing the balance between individual accuracy and diversity (Xu and Zhang, 2024). A novel evolutionary ensemble algorithm called EVINCI was proposed to provide a unique idea for solving the multiclassification imbalanced and class overlap problems simultaneously (Fernandes and de Carvalho, 2019). HCE-MCD utilizes genetic algorithms to select the optimal combination of clustering algorithms from multiple clustering algorithms and fuses the clustering results through majority voting to effectively identify and clean up overlapping region instances in multiclass datasets (Dai et al., 2024). The GAAE was used to address the issue of imbalanced healthcare data, thereby enhancing the generalization ability of classifiers (Ram and Kuila, 2023). E-MOSAIC was proposed to select the optimal combination of samples during the evolutionary process to generate high-accuracy classifiers and incorporate a diversity mechanism to improve the performance of the ensemble model (Fernandes et al., 2019). A dynamic ensemble selection (DES) strategy has been introduced for multiclass classification problems to enhance performance by dynamically selecting and optimizing classifier combinations (Zhou et al., 2017; Krawczyk et al., 2018; García et al., 2018). To further improve efficiency and handle imbalanced data, the Instance Euclidean Distance (IED) metric was developed, leading to the design of the P-EUSBagging algorithm (Pang et al., 2025). Furthermore, multiclass imbalance can be addressed using a forest of evolutionary hierarchical classifiers (FEHC), which integrates evolutionary hierarchical multiclass classifiers with SUB and STT strategies (Ning et al., 2024). Similarly, an improved evolutionary multi-objective optimization-based Bagging method was developed to enhance classification in unbalanced datasets by generating diverse and nearly balanced training sets (Roshan and Asadi, 2020). Some of the aforementioned studies have proposed a variety of effective solutions for the class imbalanced problem; however, given the complexity inherent in this kind of data, especially in multiclass scenarios, multiclass imbalanced learning is still regarded as an open problem that needs to be solved urgently (Tanha et al., 2020).

In addition, classical machine learning algorithms represented by Random Forest (Breiman, 2001) and eXtreme Gradient Boosting (Chen, 2016) are widely used in medical diagnosis. Machine learning has also demonstrated significant utility in clinical prognosis and disease diagnosis. For instance, Random Forest (RF) has been employed to predict mortality in ICU sepsis patients using the MIMIC database (Gao et al., 2024) and to assess the likelihood of successful weight loss based on early weight change characteristics (Shahabi et al., 2024). In parallel, the eXtreme Gradient Boosting (XGBoost) algorithm has been integrated with multimodal data to identify risk biomarkers for osteoarthritis (Nielsen et al., 2024), and combined with Mendelian randomization to reveal causal links between gut microbiota and cardiovascular disease (CVD) risk across diverse ethnic groups (Warmbrunn et al., 2024). Moreover, diagnostic models for various cardiovascular diseases have been constructed using routine blood and biochemical data (Wang et al., 2024), while interpretable machine learning coupled with multi-omics analysis has enabled the prediction of immunotherapy responses in cancer patients (Hounye et al., 2025). Specialized ensemble methods, such as those based on Error-Correcting Output Codes (ECOC), have also been developed for classifying angle-closure glaucoma mechanisms, providing critical support for precision medical diagnosis (Bai et al., 2016). Most of these studies used classical ensemble learning algorithms, but such algorithms are ineffective in dealing with datasets with unbalanced characteristics (Dong et al., 2020), and medical datasets are often characterized by class imbalanced, which highlights the urgency of developing new algorithms to address this challenge.

Therefore, this study constructs a new ensemble learning algorithm (Forest-EMCBE) based on genetic algorithms (Bagley, 1967), ECOC (Dietterich and Bakiri, 1994) and Bagging (Breiman, 1996), aiming at combating the multiclass imbalanced problem. The contributions of this paper are mainly in the following three aspects:

1. A novel ensemble learning algorithm, Forest-EMCBE, is proposed, which exhibits superior multiclassification performance on the CBC dataset compared with the classical ensemble learning algorithms and the imbalanced ensemble learning algorithms.
2. A new individual structure of the genetic algorithm is designed that allows for simultaneous feature selection, class reassignment and sampling and introduces an encoding mechanism that contains multiple subsets of balanced bags, which mitigates the negative impact of imbalanced class on the performance of classifiers.
3. SHAP was utilized to improve the interpretability of our model prediction results by identifying key features that influence the model’s prediction of bacterial pneumonia infection categories.

## 2 Materials and methods

### 2.1 Description and analysis

**FIGURE 1:**
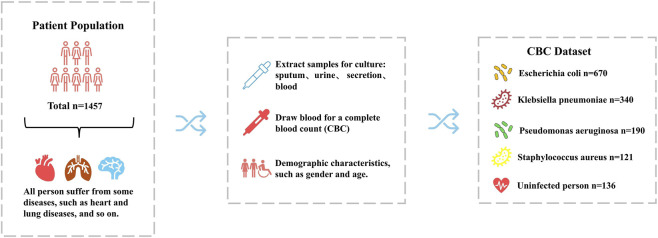
Introduction of CBC dataset. A clinical specimen was collected from each patient for bacterial culture to definitively determine the causative pathogen.

This retrospective study has been approved by the Medical Ethics Review Committee of Guangxi University. The CBC dataset analysed in this retrospective study encompassed 1,457 subjects. De-identified records were extracted from the hospital information system between 1 January and 30 June 2024 and were locked for research use on 30 September 2024. In accordance with the institutional privacy policy, no researcher had access to variables that could re-identify individual participants at any stage of the analysis. All enrolled patients had concurrent comorbidities known to predispose to bacterial pneumonia; consequently, diagnostic bacterial cultures were routinely obtained to define the aetiological agent and to inform targeted antimicrobial therapy. The following steps are usually required to determine the species of infection (Figure 1): First, samples are collected from different parts of the body for bacterial culture, including sputum, urine, secretion, and blood, depending on the type of disease the subject is suffering from. After all the samples were cultured for bacteria, five categories were obtained: 670 cases of *Escherichia coli*, 340 cases of *Klebsiella pneumoniae*, 190 cases of *Pseudomonas aeruginosa*, 121 cases of *Staphylococcus aureus*, and 126 cases from the population that were not infected with bacteria. And All four of these pathogens are conditional pathogens, which can live in symbiosis with their hosts under normal conditions without causing disease, but under specific conditions (e.g., decreased immunity, dysbiosis, and invasion of non-normal sites), they will exhibit pathogenicity.

Patients experience changes in blood composition levels following bacterial infection. Therefore, in order to investigate the intrinsic relationship between different bacterial infections, human blood composition and related self-conditions, the dataset used in this study incorporates the results of complete blood count (CBC) tests for all subjects as well. The resulting dataset integrates demographic characteristics of age and gender, multidimensional clinical characteristics of 26 CBC parameters (including white blood cell count, erythrocyte lineage markers, and so on), and specimen type. Among them, the 26 CBC parameters served as continuous features (Table 1), and age, sex (0 for female, 1 for male), and specimen type (0 for sputum, 1 for urine, 2 for secretion, 3 for blood) were discrete features (Table 2). There were a small number of missing values in the dataset, which we populated using the mean values of the corresponding feature values. The integration of these data provides a solid foundation for a comprehensive analysis of the relationship between species infection and changes in blood composition, which helps to provide an in-depth understanding of the impact of different species of infections on the hematological characteristics of patients and also provides a scientific basis for the optimization of clinical diagnosis and therapeutic strategies.

**TABLE 1:** Information about blood indicators.

Indicator	Mean	Median (Range)	Variance
WBC	9.02	7.97 (0.61–42.84)	21.75
NEUT#	7.28	5.65 (0.01–51.4)	23.46
LYMPH	1.56	1.35 (0.05–16.8)	9.68
MONO#	0.53	0.46 (0.00–2.70)	0.11
EO#	0.09	0.04 (0.00–5.00)	0.03
BASO#	0.01	0.01 (0.00–0.59)	0.0004
NEUT%	72.31	73.81 (1.60–97.30)	229.11
LYMPH%	19.73	17.02 (1.10–91.80)	166.22
MONO%	6.36	6.30 (0.00–42.00)	10.95
EO%	1.20	0.60 (0.00–19.20)	3.49
BASO%	0.14	0.1 (0.00–3.20)	0.03
RBC	4.27	4.32 (1.52–37.20)	1.34
HGB	124.78	128 (45.00–205.00)	548.82
HCT	37.31	37.90 (14.50–404.00)	139.60
MCV	87.37	87.40 (62.80–119.30)	30.17
MCH	31.52	29.60 (3.70–283.70)	54.66
MCHC	336.81	337.00 (251.00–452.00)	240.80
RDW-SD	43.97	43.00 (33.00–81.00)	37.07
RDW-CV	13.91	13.00 (11.00–27.00)	4.46
PLT	227.63	217.00 (5.00–721.00)	879.96
MPV	9.76	9.70 (0.00–13.40)	1.65
P-LCR	23.32	22.60 (0.00–50.80)	61.62
PCT	0.22	0.22 (0.00–0.67)	0.008
PDW	10.94	10.60 (0.00–98.00)	10.13
IG%	0.53	0.30 (0.00–12.60)	0.86
IG#	0.06	0.03 (0.00–2.16)	0.02
NLR	8.07	4.39 (0.01–208.1)	129.12

**TABLE 2:** Information about demographic and clinical specimen characteristics.

Variable	Value
Age	68.5 ± 14.2 (3–99)
Sex	
Female	776 (53.3%)
Male	681 (46.7%)
Specimen type	
Sputum	539 (37.0%)
Urine	520 (35.7%)
Secretion	223 (15.3%)
Blood	175 (12.0%)

### 2.2 Forest-EMCBE and a three-layer ensemble classifier

**FIGURE 2:**
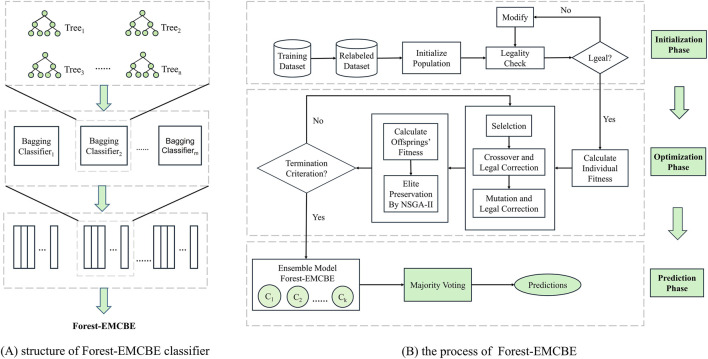
The Forest-EMCBE algorithm. **(A)** shows the three-tier integrated architecture of Forest-EMCBE classifier. **(B)** shows the evolutionary process of elite individuals within Forest-EMCBE.

The main purpose of Forest-EMCBE is to find a set of ECOC-based classifiers with higher generalization ability through genetic algorithm to form a forest of classifiers in order to counteract the problem of multiclass imbalance in the dataset. The specific process is as follows (Figure 2B), the dataset is relabeled and the individuals in the population are initialized, and then the population is evolved through the iterative process of selection, crossover, mutation, etc. At the end of the iteration, the corresponding ECOC classifiers will be trained according to the structure of each individual, and then the ensemble forest is formed.

Eventually, our model will be composed of a 3-layer integrated structure (Figure 2A). The structure of the model is Decision Trees, Bagging Classifiers, and ECOC Classifiers” from the bottom up: Layer 1 decides the prediction result by majority voting of the decision trees; layer 2 predicts the result by calculating the Euclidean distance between the predicted code and the coding of each class in the ECOC matrix. The class with the smallest distance is chosen as the prediction; layer 3 integrates the classifiers by using the F1-score of all ECOC classifiers on the validation set as weights for voting, thereby providing the final prediction for the unknown samples.

### 2.3 Individual structure

Like OvO (Zhou et al., 2017) and OvR (Ramírez et al., 2018), ECOC can exacerbate class imbalance when handling multiclass imbalanced problems (Lango and Stefanowsk, 2022). Drawing on the evolutionary ECOC (Zhang et al., 2020), this paper proposes an improved ECOC method incorporating balanced sampling, which is achieved by designing an algorithm that contains a triple genetic structure (feature selection, class reassigment, and sampling) of chromosomes to improve the generalization ability of ECOC classifiers. Among them, the bag group genes effectively mitigates the class imbalanced problem that may be exacerbated by ECOC during class reassignment.

**FIGURE 3:**
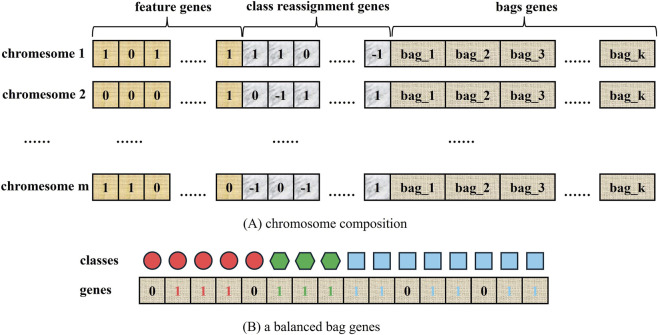
Individual structure. **(A)** shows that each individual consists of several chromosomes, each of which is composed of three types of genes: feature genes, class-reassignment genes, and bag genes. **(B)** shows that after sampling via the bag genes, the sample counts across all classes are automatically balanced.

Each individual consists of a number of chromosomes (Figure 3A), which are composed of feature selection genes, class reassignment genes and bag group genes, and the roles of each part of the genes are as follows:

1. Feature selection genes: it employs a number of binary codes, where 1 bit of 1/0 indicates that the corresponding characteristic is selected/unselected;
2. Class reassignment genes: it contains a number of three-valued bits to represent a class reassignment scheme, where the classes corresponding to 0 is not involved in the training process, and the classes corresponding to 1/-1 are converted into positive/negative class. In this way, a multiclass problem is transitively converted into a binary classification problem.
3. Bag group genes (Figure 3B): it contains k balanced training subsets, each of which keeps the classes balanced by randomly sampling. The reassignment genes for the three classes are {1, 1, −1}, and their sample sizes are {5, 3, 8}. Therefore, class 0 and class 1 are converted to positive classes, and class 2 is converted to negative classes, and to ensure that the training subset is balanced, the number of samples for class 0, class 1, and class 2 will be 3, 3, and 6, which creates a balanced training subset for binary classification. Since classical machine learning algorithms directly train classifiers on imbalanced datasets, causing the classifiers to favor predicting the majority class to improve overall accuracy. However, in our approach, using multiple balanced training subsets fundamentally mitigates the negative effects of class imbalance in the original dataset. Multiple bags will produce multiple balanced training subsets to construct diverse decision trees, which are then integrated to form a Bagging classifier. To validate the effectiveness of this structure, this study sets up two types of individuals to produce two ensemble classifiers. The first type consists of feature selection and class reassignment genes, and the ensemble model generated is used as a baseline for comparison; the second class contains three genes, with more bag genes than the first class, and the model generated is called Forest-EMCBE. The two models will be compared in the experimental session.

### 2.4 Initialization of population

Based on the individual structure, the population initialization process is as follows: first, the class label redistribution mechanism is used to relabel each class in descending order according to the sample size of every class. Assuming that the original sample number of class 0 to 2 in the dataset is {10, 20, 30}, the class labels will be relabeled as 2 to 0, and the instances of each class will be arranged in order, which is conducive to the subsequent sampling. Then, initialize the individuals in the population: generate feature selection and class reassignment genes, and calculate the number of samples in each balanced subset based on the latter; finally, perform random sampling to construct a balanced training set and train an ensemble binary classifier to evaluate the fitness of individuals in the initial population. Superior to the Boosting algorithm (Mayr et al., 2014), Bagging algorithm is able to produce classifiers with better generalization ability in most cases (Khoshgoftaar et al., 2010); therefore, this method is used to generate a Bagging classifier for each column of the ECOC matrix to solve the task of the binary classification problem after class reassignment.

### 2.5 Fitness function

In genetic algorithms (GAs), the fitness function is important in affecting the evolutionary direction of the entire population. In our GA, the F1 score (Equation 3) is used as the first optimization objective of the individual. It is an effective metric for evaluating classifier performance on imbalanced datasets and is calculated from precision (Equation 1) and recall (Equation 2). Another key to determining the performance of the ensemble classifier is the diversity among the base classifiers (Cruz et al., 2018), which refers to the difference between the predictions produced by the different base classifiers (Mao et al., 2019); therefore, the Prediction Diversity (Equation 4) is used as the second objective of an individual. ,m denotes the number of individuals in thr population, i and j denote the *i*th and *j*th individuals, N denotes the number of samples in the sample set, o_i,n_ denotes the prediction result of the *i*th individual for the *n*th sample, and the expression |o_i,n_-o_j,n_| denotes whether individuals i and j predicted correctly or incorrectly for the *n*th sample at the same time and returns 0 if it is true and returns 1 otherwise.

The performance of individual on the two objectives together determines the individual’s fitness value to decide whether the individual can proceed to the next round of the iterative process.

$$\text{precision}=\text{avg}\left(\sum_{i=1}^{n}\frac{TP_{i}}{TP_{i}+FP_{i}}\right).$$ (1)

$$\text{recall}=\text{avg}\left(\sum_{i=1}^{n}\frac{TP_{i}}{TP_{i}+FN_{i}}\right).$$ (2)

$$F1-\text{score}=\text{avg}\left(\sum_{i=1}^{n}\frac{2\cdot {\text{precision}}_{i}\cdot {\text{recall}}_{i}}{{\text{precision}}_{i}+{\text{recall}}_{i}}\right).$$ (3)

$$PD_{i}=\frac{\sum_{\forall j\in \left[1,m\right],i\neq j}\left(\sum_{n=1}^{N}\left|o_{i,n}-o_{j,n}\right|\right)}{m-1}.$$ (4)

### 2.6 The process of selection and reproduction

At the beginning of each iteration, we used the tournament selection method to select the parent. Specifically, 2 parents are selected from each of the 3 randomly selected individuals, and the individual with the higher F1-score is selected as the parent, and if there is a tie in F1-score, the judgment is based on the second target value.

**FIGURE 4:**
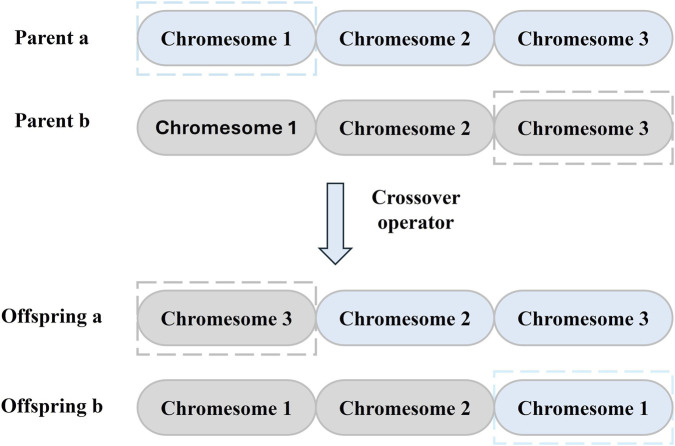
An example of crossover operation. Each parent contributes one chromosome to a reciprocal exchange, yielding two recombinant offspring.

The detailed mechanism of the crossover process (Figure 4) ensures the exchange of genetic information between parent individuals. After completing the selection of parents, the crossover operator is applied to produce 2 offspring, which in total will produce a number of offspring comparable to the number of the initial population, and the crossover operation is to exchange a random chromesome of the two parents. Individuals after crossover have a probability of mutation, which helps to prevent the algorithm from falling into a local optimum.

**FIGURE 5:**
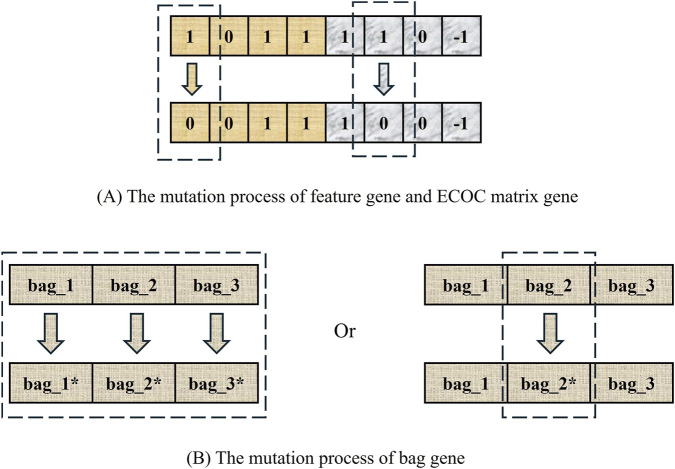
An example of mutation operation. **(A)** shows that a feature gene and a class-reassignment gene will each undergo single-bit flipping. **(B)** shows that the two variation modes of the bag genes.

The mutation process is illustrated (Figure 5), involving distinct operations for different gene types. In the case of feature selection genes, one gene will be randomly selected for bit-flip operation; for class reassignment genes, the probability of mutation occurring is 0.5, and one gene will be randomly mutated, specifically changing −1 to 1, 1 to −1, or 0 to −1 or 1; in the case of bag group genes, there are two ways of mutation; if the class reassignment genes mutate, all bag genes need to be randomly re-sampled because the bag genes are affected by them. If the class reassignment gene is not mutated, only the gene of one bag needs to be randomly replaced.

### 2.7 Elitism strategy and termination criteria

We used the elite retention strategy of NSGA2 (Deb et al., 2002) to select individuals to enter the next iteration. After completing the selection, crossover, and mutation operations, all individuals (including parents and offspring) are ranked by fitness using fast non-dominated sorting, and new populations with the same size as the initial population are subsequently selected based on the ranking from highest to lowest to enter the next iteration. The iteration termination condition sets a fixed maximum number of iterations.

## 3 Results

### 3.1 Experimental settings

In the experiments, the relevant hyperparameters of the algorithms are set as follows: The population size is 60; the maximum number of iterations is 30; the number of bags k in the bag group is 9; the crossover probability is 1; the initial mutation probability is 0.01; the number of columns of the ECOC coding matrix is between [n, 3*n - 1], where n denotes the number of classes; and the number of base classifiers is half of the number of individuals in the Pareto first frontier. All hyperparameter selections follow empirical settings widely adopted in existing literature (Fernandes et al., 2019; Xu and Zhang, 2024).

All comparison experiments are analyzed based on five-fold cross-validation, and the results of all algorithms are averaged over 20 independent runs, and the effectiveness of each algorithm is assessed by F1 score (Equation 3), G-mean (Equation 5), and precision metrics (Equation 1). They are both valid measures of model performance in datasets with imbalanced classes.

$$G-\text{mean}={\left(\prod_{i=1}^{n}{r\text{ecall}}_{i}\right)}^{\frac{1}{n}}$$ (5)

As described in Section 2.3, our algorithm consists of two versions: the Baseline model is an ECOC forest that simply integrates decision trees with only two layers of integration structure (Decision Trees-ECOC Classifiers); the Forest-EMCBE model is an ensemble forest with three layers of ensemble structure (Decision Trees-Bagging Classifiers-ECOC Classifiersrs). In the experimental section, their performances will be compared to verify the effectiveness of the individual “bag group gene” in the genetic algorithm.

In addition, in this study, SHAP values were used in the prediction results of our model to clarify the degree of contribution of each feature to the prediction results of bacterial categories. Through the SHAP analysis method, we were able to quantify the contribution of each feature to the model predictions, as well as visualize the differential impact of each feature on the model predictions of different bacterial pneumonia.

To investigate whether performance differences among selected algorithms are statistically significant, rigorous statistical methods must be employed to analyze experimental results. This study utilizes the Wilcoxon signed-rank test for pairwise comparisons to explicitly identify significant differences between specific algorithm pairs. All statistical tests are conducted at a 95% confidence level.

### 3.2 Model performance

#### 3.2.1 Ablation study

**FIGURE 6:**
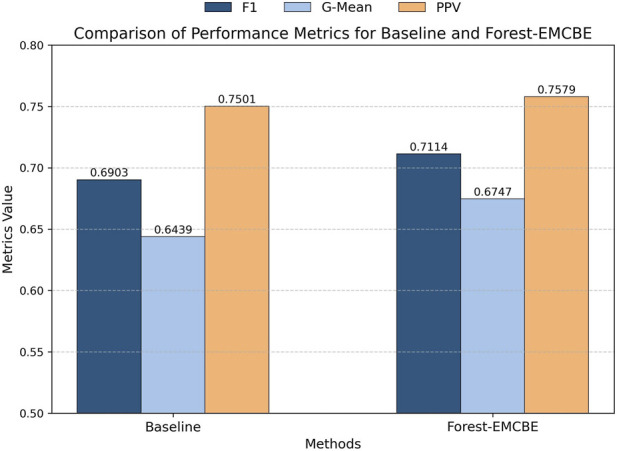
Forest-EMCBE was compared with the baseline model, which evolved from individuals without the bag group genes. This comparison demonstrates the effectiveness of bag group genes in enhancing model performance.

The performance of the baseline model is compared with the Forest-EMCBE model across three metrics (Figure 6). As can be seen from the figure, the Forest-EMCBE model outperforms the baseline model in all three performance metrics. Specifically: in terms of F1-score, the baseline model has an F1-score of 0.6903, while the Forest-EMCBE model has an F1-score of 0.7114, which is an improvement of 2.11%; in terms of G-mean value, the baseline model has a G-mean value of 0.6439, while the Forest-EMCBE model has a G-mean value of 0.6747, which is an improvement of 3.08%; in terms of precision value, the baseline model has a precision value of 0.7501, and the Forest-EMCBE model has a precision value of 0.7579, which is an improvement of 0.78%.

To further validate the performance advantages of Forest-EMCBE, we conducted a statistical comparison using the Wilcoxon signed-rank test (Table 3). Forest-EMCBE significantly outperformed the baseline model on both G-mean and F1-score metrics. Regarding the Precision metric, no statistically significant difference was observed between the two models (p-value = 5.68 × 10^−1^).

**TABLE 3:** Results of Wilcoxon tests for comparing Forest-EMCBE with baseline.

Performance measure	Comparsion	Hypothesis	p-value
G-mean	Baseline	Rejected at 5%	1.56 × 10^−11^
F1-score	Baseline	Rejected at 5%	5.20 × 10^−10^
Precision	Baseline	Not rejected	5.68 × 10^−1^

Overall, compared with the baseline model, the Forest-EMCBE model has more obvious advantages in the F1-score and G-mean value, which fully verifies the effectiveness of the bag group genes in facing the multiclass imbalanced problem.

#### 3.2.2 Compared with imbalanced ensemble learning algorithms

**FIGURE 7:**
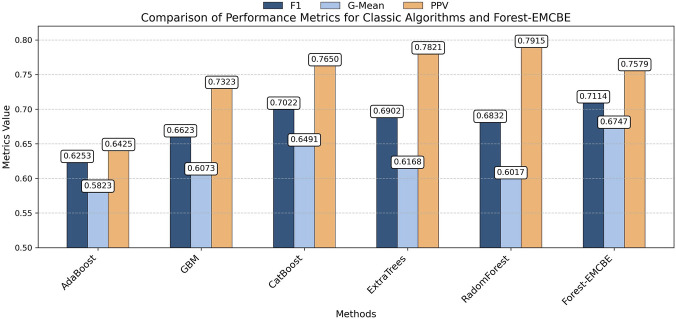
Forest-EMCBE compared with Imbalanced Ensemble Learning Algorithms.

Six imbalanced ensemble learning algorithms are used for comparison with our algorithm, including SMOTEBoost, SMOTEBagging, RUSBoost, BalancedRF, EasyEnsemble, and FEHC (Figure 7). Among them, The first five algorithms are all from the imbens package, which focuses on solving the class imbalanced problem, while the FEHC algorithm, proposed in 2024, is a novel ensemble learning algorithm for dealing with multiclass imbalanced problems (Ning et al., 2024). To ensure the fairness of the experiments, all algorithms use decision trees as base classifiers algorithms.

The details are as follows:

1. In terms of F1 score, the SMOTEBoost, BalancedRF, RUSBoost, EasyEnsemble, FEHC, and SMOTEBagging methods have F1-score of 0.6747, 0.5965, 0.6271, 0.6278, 0.6613, and 0.6980, respectively. The Forest-EMCBE method had the highest F1-score of 0.7114, an improvement of about 1.8% compared to the next best-performing SMOTEBagging and about 6% higher than the average of these methods.
2. In terms of G-mean values, the methods had G-mean values of 0.6479, 0.6551, 0.6271, 0.6549, 0.6971, and 0.6612, respectively. The Forest-EMCBE method had a G-mean value of 0.6747, which was only about 1.1% lower than the optimal FEHC but about 2% higher than the average of these methods.
3. In terms of precision values, the precision values of the methods are 0.6994, 0.5795, 0.6150, 0.6159, 0.6494, and 0.7337, respectively. The Forest-EMCBE method has the highest precision value of 0.7579, which is improved compared to the second best-performing SMOTEBagging method by 2.42% and is about 10% higher than the average of these methods.

To statistically validate the significance of performance differences between Forest-EMCBE and other imbalanced ensemble learning algorithms, we employed the Wilcoxon signed-rank test for paired comparisons. Statistical results indicate that Forest-EMCBE demonstrated statistically significant advantages (p < 0.05) over all comparison methods in F1-score and Precision metrics (Table 4). Regarding the G-mean metric, Forest-EMCBE also significantly outperformed the other five methods, except for the difference with FEHC.

**TABLE 4:** Results of Wilcoxon tests for comparing Forest-EMCBE with imbalanced ensemble learning algorithms.

Performance measure	Comparsion	Hypothesis	p-value
G-mean	BalancedRF	Rejected at 5%	2.32 × 10^−2^
	EasyEnsemble	Rejected at 5%	7.07 × 10^−4^
	FEHC	Rejected at 5%	6.36 × 10^−5^
	RUSBoost	Rejected at 5%	2.75 × 10^−12^
	SMOTEBagging	Rejected at 5%	6.40 × 10^−4^
	SMOTEBoost	Rejected at 5%	6.99 × 10^−6^
F1-score	BalancedRF	Rejected at 5%	4.67 × 10^−18^
	EasyEnsemble	Rejected at 5%	9.60 × 10^−18^
	FEHC	Rejected at 5%	3.90 × 10^−14^
	RUSBoost	Rejected at 5%	8.02 × 10^−18^
	SMOTEBagging	Rejected at 5%	4.36 × 10^−4^
	SMOTEBoost	Rejected at 5%	1.60 × 10^−12^
Precision	BalancedRF	Rejected at 5%	3.90 × 10^−18^
	EasyEnsemble	Rejected at 5%	4.02 × 10^−18^
	FEHC	Rejected at 5%	6.91 × 10^−18^
	RUSBoost	Rejected at 5%	4.02 × 10^−18^
	SMOTEBagging	Rejected at 5%	1.62 × 10^−4^
	SMOTEBoost	Rejected at 5%	1.42 × 10^−15^

In summary, in the comparison with the imbalanced ensemble learning algorithms, the Forest-EMCBE method achieves the best results in the F1 score and Precision metrics; It ranked second in the G-Mean metric, although it was slightly lower than FEHC, it significantly outperformed FEHC in terms of F1 score and precision Considering the performance of all the metrics, the Forest-EMCBE method shows significant advantages in the three performance indicators of F1, G-mean, and precision, especially in the improvement of the precision, which indicates that our method has high competitiveness in dealing with the multiclass imbalanced problem in the CBC dataset.

#### 3.2.3 Compared with classic ensemble learning algorithms

**FIGURE 8:**
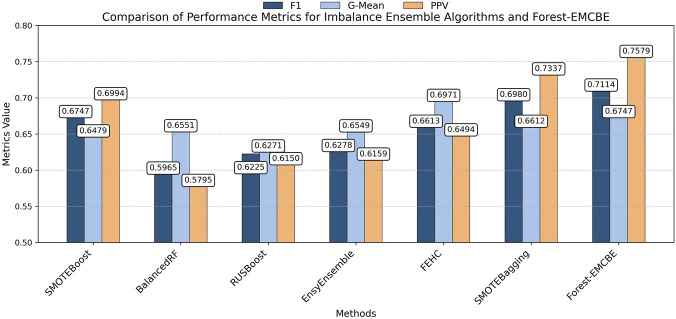
Forest-EMCBE compared with Classical Ensemble Learning Algorithms.

Meanwhile, five classical ensemble learning algorithms were used to compare with our algorithm, including AdaBoost, CatBoost, GBM, ExtraTrees, and RandomForest (Figure 8), which are widely used in healthcare datasets and are effective methods for dealing with complex classification problems. The comparison details are shown:

1. In terms of F1 score, Forest-EMCBE has the highest F1 score, which improves by about 1.4% compared to the next best-performing CatBoost method and is about 6.5% higher than the average of these methods.
2. In terms of G-mean, Forest-EMCBE also had the highest G-mean value of 0.6747, which was about 5.2% higher than the average of these methods.
3. In terms of precision, Forest-EMCBE is not as good as CatBoost, ExtraTrees, and RandomForest but is still higher than AdaBoost and GBM.

Similarly, when performing statistical analysis with classical ensemble algorithms (Table 5), Forest-EMCBE significantly outperformed all methods on the G-mean metric (p < 0.05). It also demonstrated statistically significant advantages over all methods except CatBoost on both the F1-score and Precision metrics.

**TABLE 5:** Results of Wilcoxon tests for comparing Forest-EMCBE with classic ensemble learning algorithms.

Performance measure	Comparsion	Hypothesis	p-value
G-mean	AdaBoost	Rejected at 5%	2.08 × 10^−17^
	GBM	Rejected at 5%	9.60 × 10^−16^
	CatBoost	Rejected at 5%	4.15 × 10^−6^
	ExtraTrees	Rejected at 5%	6.59 × 10^−12^
	RandomForest	Rejected at 5%	1.31 × 10^−15^
F1-score	AdaBoost	Rejected at 5%	5.27 × 10^−18^
	GBM	Rejected at 5%	2.48 × 10^−14^
	CatBoost	Not rejected	5.12 × 10^−2^
	ExtraTrees	Rejected at 5%	1.31 × 10^−4^
	RandomForest	Rejected at 5%	4.24 × 10^−7^
Precision	AdaBoost	Rejected at 5%	4.14 × 10^−18^
	GBM	Rejected at 5%	4.15 × 10^−6^
	CatBoost	Not rejected	6.34 × 10^−2^
	ExtraTrees	Rejected at 5%	1.05 × 10^−4^
	RandomForest	Rejected at 5%	2.01 × 10^−7^

In summary, in comparison with classical ensemble learning algorithms, our algorithm Forest-EMCBE is lower than CatBoost, ExtraTrees, and RandomForest in terms of precision but achieves the best results in terms of F1 score and G-mean, and the G-mean is significantly higher than that of all other algorithms. Although the precision of our method is lower than that of most classical algorithms, higher recall is often more critical in medical diagnosis, as it indicates a lower probability of missed diagnoses (Salmi et al., 2024). This is particularly vital in clinical settings where failing to identify a positive case (e.g., a disease) can lead to severe consequences for patients. Therefore, our approach prioritizes sensitivity to ensure that fewer true cases are overlooked, even at the potential cost of more false positives. Taken together, Forest-EMCBE performs better than these classical ensemble learning algorithms on the CBC dataset.

### 3.3 SHAP value analysis

#### 3.3.1 Overall feature importance analysis for each class

**FIGURE 9:**
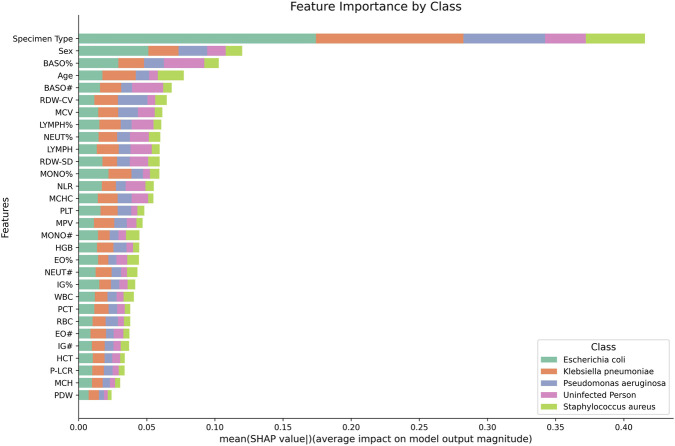
Global feature importance based on mean SHAP values across all categories. The stacked bar chart illustrates the average impact of each blood parameter on the model’s output magnitude. Each color segment represents the contribution of a specific class: *Escherichia coli*, *Klebsiella pneumoniae*, *Pseudomonas aeruginosa*, *uninfected individuals*, and *Staphylococcus aureus*.

In this study, the importance of features in the machine learning model was systematically assessed using SHAP value analysis, aiming to clarify the contribution of each feature to the model’s prediction of results for different bacteria strains. The average SHAP value of each feature is presented in the form of a horizontal bar graph, which visualizes the average degree of influence of the feature on the model output (Figure 9). The results show that the “Specimen Type” “Gender” and “BASO%” features have the highest average SHAP values, indicating that these three features have the greatest impact on model predictions.

In addition, there were significant differences in the extent to which the same feature contributed to the prediction of different bacterial categories. For example, “BASO%” was more important than “Age” in predicting *E. coli* infections and less important than “Age” in predicting *Klebsiella pneumoniae* infections. This finding reveals that the model differentially utilizes features based on their relevance to specific bacterial categories when making bacterial class predictions. This flexibility in feature selection and utilization is particularly important for medical diagnosis, as it can help physicians better understand the role and importance of different features in the diagnosis of different bacterial pneumonia.

#### 3.3.2 Feature contributions to different bacterial pneumonia prediction

**FIGURE 10:**
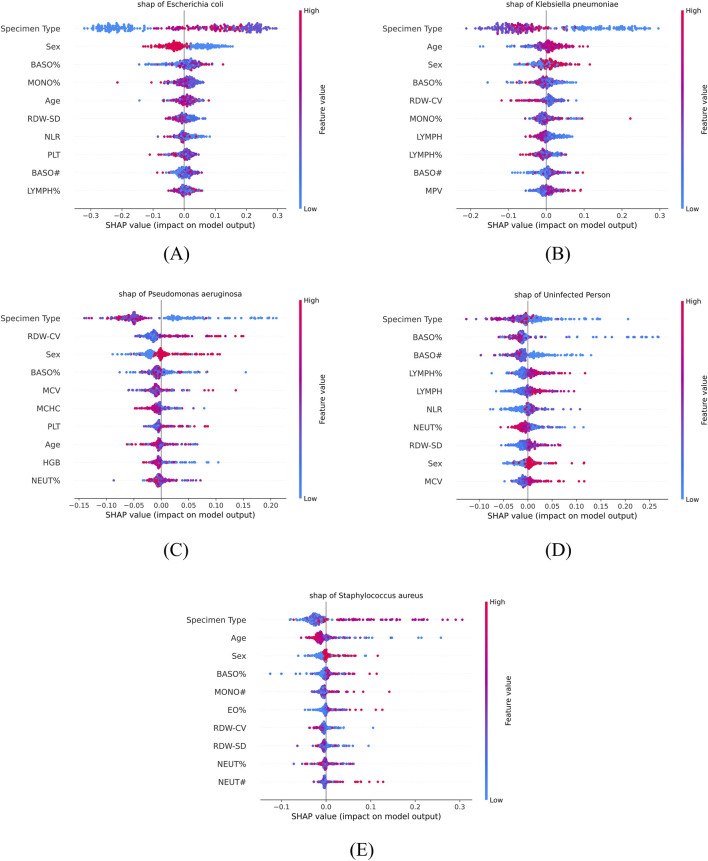
SHAP beeswarm plots for feature importance across different infection categories. The plots illustrate the impact of blood parameters on the classification of **(A)** *Escherichia coli*, **(B)** *Klebsiella pneumoniae*, **(C)** *Pseudomonas aeruginosa*, **(D)** *uninfected individuals*, and **(E)** *Staphylococcus aureus*.

**FIGURE 11:**
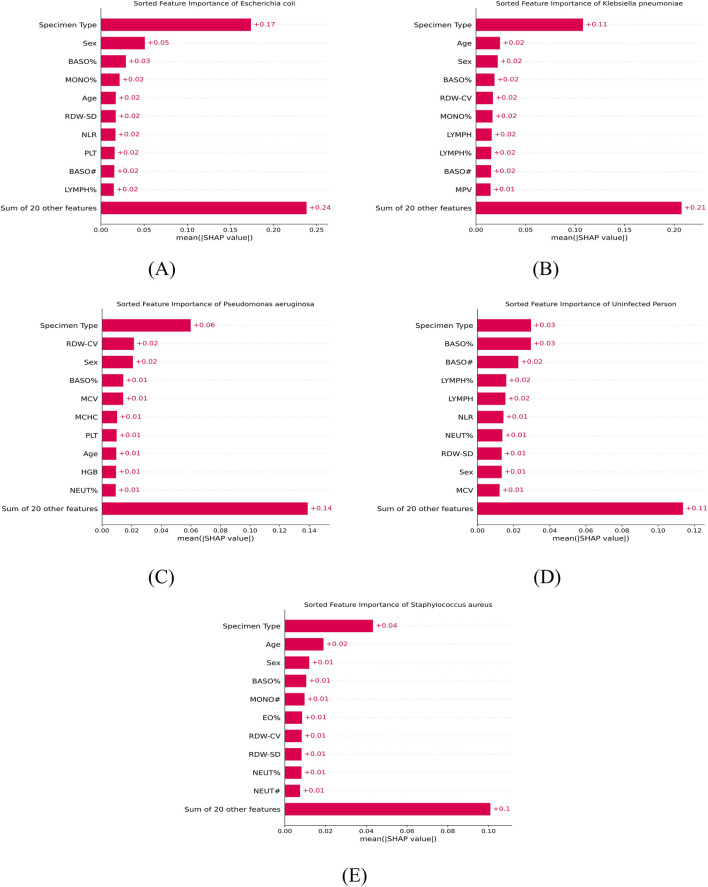
Sorted feature importance for specific infection classes and uninfected individuals. The bar charts rank the clinical features based on their mean absolute SHAP values for **(A)** *Escherichia coli*, **(B)** *Klebsiella pneumoniae*, **(C)** *Pseudomonas aeruginosa*, **(D)** *uninfected individuals*, and **(E)** *Staphylococcus aureus*. The “Sum of 20 other features” represents the combined predictive contribution of the remaining variables.

Swarm and bar charts (Figures 10, 11) are utilized to illustrate the contribution of various features to the model’s predictive outcomes, with the top 10 most influential features highlighted. We find that the feature “Specimen Type” contributes the most to the model’s prediction of all five classes, and the type of specimens selected correlates with the original disease that the patient suffered from, which may imply that people suffering from a specific disease are more susceptible to a particular type of specific bacterial pneumonia. There also appears to be a predilection for the sex of the infected individuals, with females (sex = 0) having a higher risk of infection for *Escherichia coli* and males (sex = 1) having a higher risk of infection for *Klebsiella pneumoniae*, *Pseudomonas aeruginosa*, and *Staphylococcus aureus*. For each bacterial species, the top five features influencing the prediction were identified as follows:

1. *Escherichia coli*: Specimen type, sex, BASO%, MONO%, and age. Higher BASO% and lower MONO% increased the predicted probability, while older individuals were more likely to be infected.
2. *Klebsiella pneumoniae*: Specimen type, age, sex, BASO%, and RDW-CV. Lower BASO% and RDW-CV increased the predictive probability, and older individuals were more likely to be infected.
3. *Pseudomonas aeruginosa*: Specimen type, RDW-CV, sex, BASO%, and MCV. Higher RDW-CV and MCV, as well as lower BASO%, increased the predictive probability.
4. *Staphylococcus aureus*: Specimen type, age, sex, BASO%, and MONO#. Younger individuals were more likely to be infected, and higher BASO% and MONO# enhanced the model’s prediction of the probability of infection.

#### 3.3.3 Individual-level analysis of infection prediction

**FIGURE 12:**
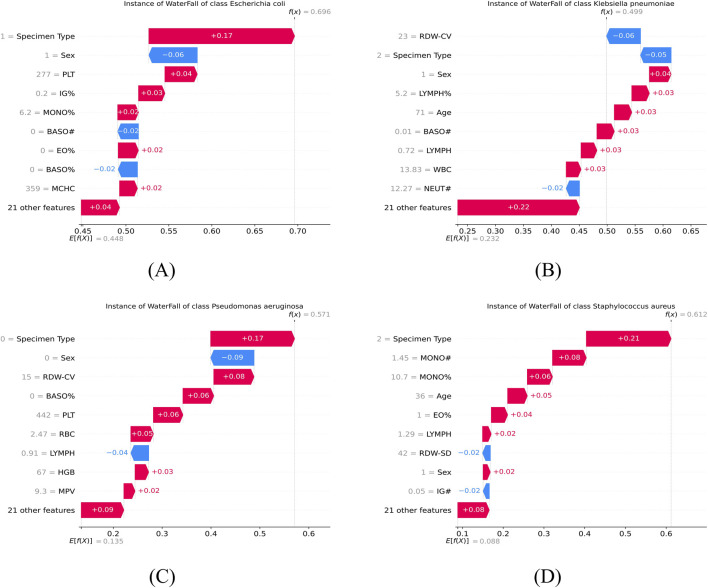
SHAP waterfall plots for individual sample interpretation. The plots provide a local explanation of the model decision process for representative cases of **(A)** *Escherichia coli*, **(B)** *Klebsiella pneumoniae*, **(C)** *Pseudomonas aeruginosa*, and **(D)** *Staphylococcus aureus*. Red bars indicate features that increase the prediction probability, while blue bars indicate features that decrease it.

Waterfall plots using SHAP values allowed us to quantify the impact of individual patient data on the types of bacterial pneumonia predicted by the model. We extracted and used waterfall plots to visualize the predicted positive and negative effects of different individuals infected with different bacteria. For instance, the risk profile of a patient predicted to have an *Escherichia coli* infection (Figure 12A) is primarily driven by specific blood metrics, such as PLT and Specimen Type. However, the gender (being male) of this patient had a negative impact on the prediction, reducing the probability of being predicted to have an *Escherichia coli* infection. Other features such as MONO% EO% also had smaller positive or negative contributions.

In this way, physicians are able to understand the changes in blood composition of a patient after an infection on an individual level and are able to identify which features have the greatest impact on the prediction of a particular infection type, thus providing support for clinical decision-making. This individualized analysis helps physicians understand each patient’s condition more precisely and optimize treatment options.

## 4 Discussion

Our study was carried out based on CBC dataset and machine learning algorithms, and significant progress was made in constructing a fast and accurate diagnostic model for bacterial pneumonia. In terms of model performance, our Forest-EMCBE model achieves 0.7114, 0.6747, and 0.7579 in F1-score, G-mean, and precision, respectively, which is better compared to other algorithms of the same type. This indicates that our model has a better classification effect on the CBC dataset and can better cope with the multiclass imbalanced problem existing in the dataset. In terms of feature importance analysis, we introduced SHAP values for analysis to visualize the differential impact of every features on the prediction of different types of bacterial pneumonia and identified key features such as Specimen Type, Sex, BASO%, age, and so on that have a greater impact on the diagnosis of bacterial pneumonia.

Nevertheless, our model has demonstrated improvements in both performance and predictive accuracy; however, this study still has several limitations. The dataset used is relatively small in sample size, which may constrain the model’s generalizability, and no additional independent datasets were available for external validation. More importantly, all data are derived from the same batch of standardized samples, and the model’s generalizability across different clinical centers, diagnostic platforms, or reagent batches has not yet been validated. Furthermore, the evolutionary process of the Forest-EMCBE algorithm is computationally complex, resulting in high training time overhead. In future work, we plan to expand the dataset and optimize the algorithm, with the goal of developing more generalizable and accurate diagnostic models for bacterial pneumonia identification.

## Data Availability

The raw data supporting the conclusions of this article will be made available by the authors, without undue reservation.
